# Functionalization Strategies of Chitosan-Based Scaffolds with Growth Factors for Bone Regeneration: A Systematic Review

**DOI:** 10.3390/md23100396

**Published:** 2025-10-09

**Authors:** Jan Kiryk, Mateusz Michalak, Zuzanna Majchrzak, Marzena Laszczyńska, Sylwia Kiryk, Sylwia Szotek, Hanna Gerber, Izabela Nawrot-Hadzik, Jacek Matys, Maciej Dobrzyński

**Affiliations:** 1 Dental Surgery Department, Wroclaw Medical University, Krakowska 26, 50-425 Wroclaw, Poland; jan.kiryk@umw.edu.pl; 2 Medical Center of Innovation, Wroclaw Medical University, Krakowska 26, 50-425 Wroclaw, Poland; mateusz.michalak92@gmail.com (M.M.); zuzanna.h.nawrocka@gmail.com (Z.M.);; 3 Department of Pediatric Dentistry and Preclinical Dentistry, Wroclaw Medical University, Krakowska 26, 50-425 Wroclaw, Poland; s.roguzinska@gmail.com (S.K.); maciej.dobrzynski@umw.edu.pl (M.D.); 4 Department of Mechanics, Materials and Biomedical Engineering, Faculty of Mechanical Engineering, Wroclaw University of Science and Technology, Lukasiewicza 7/9, 50-371 Wroclaw, Poland; sylwia.szotek@pwr.edu.pl; 5 Department of Maxillofacial Surgery, Wroclaw Medical University, Borowska 213, 50-556 Wrocław, Poland; hanna.gerber@umw.edu.pl; 6 Department of Pharmaceutical Biology and Biotechnology, Faculty of Pharmacy, Wroclaw Medical University, 50-556 Wroclaw, Poland; izabela.nawrot-hadzik@umw.edu.pl

**Keywords:** BMP-2, bone regeneration, chitosan scaffold, growth factor, VEGF

## Abstract

Bioactive agents can stimulate osteogenesis, angiogenesis, and cell proliferation; therefore, their application in bone regeneration offers significant therapeutic potential. The aim of this systematic review was to evaluate strategies for applying chitosan-based scaffolds with growth factors in bone regeneration. A structured literature search was conducted in July 2025 across the PubMed, Scopus, and Web of Science databases. Search terms included combinations of (chitosan scaffold) AND (growth factor OR BMP-2 OR VEGF OR FGF OR TGF-beta OR periostin OR PDGF OR IGF-1 OR EGF OR ANG-1 OR ANG-2 OR GDF-5 OR SDF-1 OR osteopontin). The study selection process followed PRISMA 2020 guidelines and the PICO framework. Out of 367 records, 226 were screened, and 17 studies met the eligibility criteria for qualitative analysis. BMP-2 was the most frequently investigated growth factor, studied in both in vitro and in vivo models, with rats and rabbits as the most common animal models. Scaffold compositions varied, incorporating hydroxyapatite, heparin, polyethylene glycol diacrylate, octacalcium phosphate-mineralized graphene, silk fibroin, and aloe vera. Growth factors were introduced using diverse methods, including microspheres, chemical grafting, covalent coupling, protein carriers, and nanohydroxyapatite mesopores. Most studies reported enhanced bone regeneration, although differences in models, scaffold composition, and delivery methods preclude definitive conclusions. The addition of growth factors generally improved osteoblast proliferation, angiogenesis, bone density, and expression of osteogenic markers (RunX2, COL1, OPN, OCN). Combining two bioactive agents further amplified osteoinduction and vascularization. Sustained-release systems, particularly those using heparin or hydroxyapatite, prolonged biological activity and improved regenerative outcomes. In conclusion, functionalization of chitosan-based scaffolds with growth factors shows promising potential for bone regeneration. Controlled-release systems and combinations of different bioactive molecules may offer synergistic effects on osteogenesis and angiogenesis. Further research should focus on optimizing scaffold compositions and delivery methods to tailor bioactive agent release for specific clinical applications.

## 1. Introduction

Bone regeneration remains a major clinical challenge, particularly in critical-sized defects (>2 cm) resulting from severe trauma, oncological resection, osteomyelitis, or congenital anomalies, where endogenous healing alone cannot restore tissue continuity [1,2,3,4,5,6,7,8]. Conventional treatments, such as autologous, allogeneic, or xenogeneic bone grafts, face substantial limitations, including donor-site morbidity, limited availability, immunogenic reactions, and infection risk [2,3,9,10,11,12]. Although synthetic bone substitutes are standardized and commonly available, they often lack the hierarchical structure and essential bioactivity required for complete tissue regeneration [1,13,14,15,16,17,18,19,20]. Moreover, large bone defects are frequently characterized by insufficient vascularization and poor recruitment of osteoprogenitor cells, further inhibiting the repair process [2,9,11]. These challenges underscore the need for precision-engineered biomaterials that closely mimic extracellular matrix architecture, promote both angiogenesis and osteogenesis, and enable controlled delivery of bioactive agents. Multidisciplinary strategies that integrate advanced scaffold design, growth factor-based release systems, and recruitment of endogenous stem cells or progenitor cells are therefore essential to overcome current clinical barriers and achieve functional bone regeneration [2].

**Figure 1:**
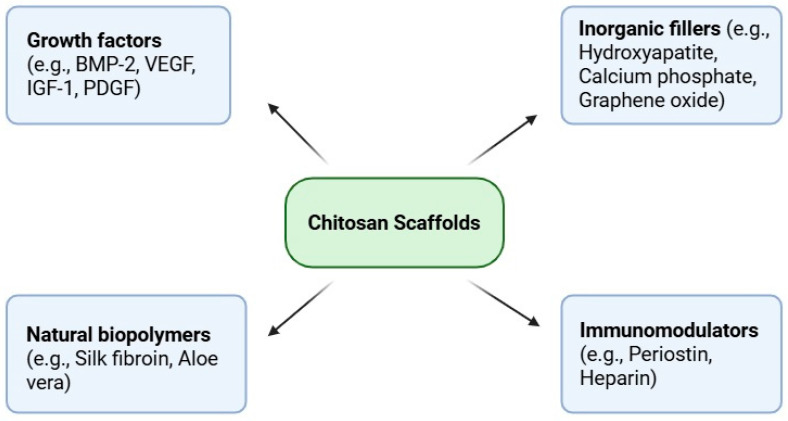
Representative categories of bioactive agents incorporated into chitosan-based scaffolds for bone regeneration, including growth factors, inorganic fillers, natural biopolymers, and immunomodulators.

Chitosan is a linear polysaccharide derived from chitin. It consists of deacetylated units (β-1,4-D-glucosamine) and residual acetylated units (2-acetamido-2-deoxy-β-D-glucose). It exhibits excellent biocompatibility, biodegradability, antimicrobial properties, and strong support for cell adhesion and proliferation, making it one of the most extensively investigated scaffold materials in bone tissue engineering [3,13,21,22,23,24,25,26,27,28,29,30,31]. However, unmodified chitosan lacks osteoinductive properties, which cause the need for functionalization strategies to promote bone formation in situ [32]. Chemical modifications such as phosphorylation or sulfation enhance BMP-2 binding and stimulate osteogenic differentiation of mesenchymal stem cells [2,13,33]. Composite scaffolds that integrate chitosan with inorganic nanomaterials—including nanohydroxyapatite, bioactive glass, or graphene oxide—have been shown to improve mechanical strength, support mineralization, and increase bone volume in critical-sized defect animal models [1,34,35,36,37,38]. Moreover, chitosan-based biomaterials influence the immune microenvironment by modulating macrophage polarization toward a pro-regenerative M2 phenotype, thereby accelerating bone repair [21,39,40,41]. Collectively, while chitosan represents a comprehensive scaffold platform, its full regenerative potential is achieved through synergistic combination with osteoinductive agents, mineral reinforcements, and immunomodulatory design [13,21,22,26,42] (Figure 1).

**Figure 2:**
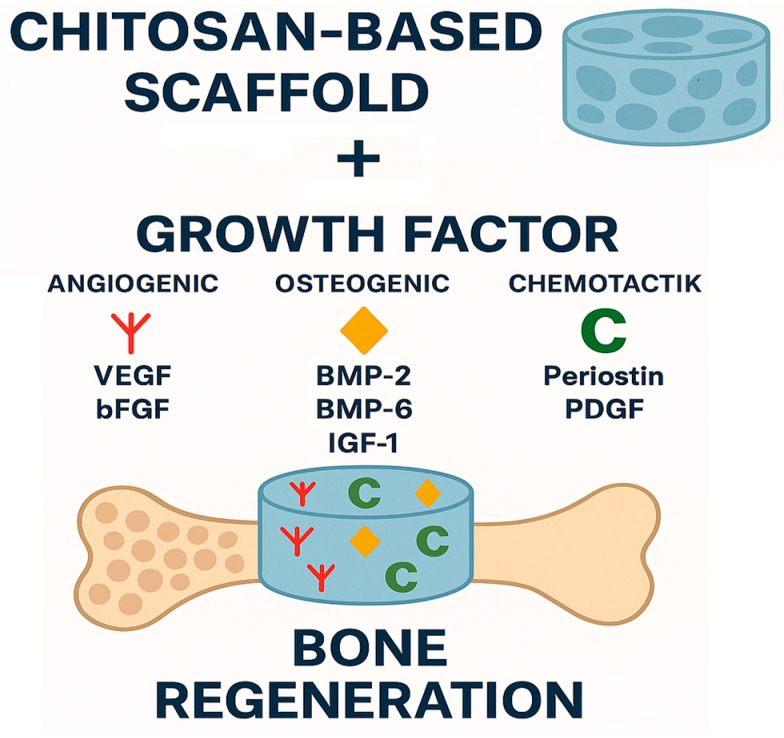
Schematic representation of bone regeneration using a chitosan-based scaffold combined with growth factors. A porous chitosan scaffold serves as the carrier for three categories of growth factors: angiogenic (VEGF, bFGF), osteogenic (BMP-2, BMP-6, IGF-1), and chemotactic (periostin, PDGF). Their combined action enhances vascularization, cell recruitment, and osteogenesis, thereby promoting effective bone regeneration.

The functionalization of chitosan scaffolds is a rational design strategy aimed at enhancing their biological performance by incorporating growth factors into the scaffold structure. The choice of bioactive agent—such as BMP-2, BMP-6, VEGF, PDGF, IGF-1, or bFGF—depends on the intended therapeutic outcome, whether to stimulate osteogenesis, promote angiogenesis, or enhance cellular proliferation [43,44,45,46,47,48] (see Figure 2). Equally important is the method of incorporation, which directly influences release kinetics and biological activity [49,50]. Chemical grafting (e.g., P24 from BMP-2 onto CS–HA scaffolds [51]) and covalent coupling (BMP-2 linked to PEG diacrylate–CS scaffolds [52,53]) support strong cell adhesion, proliferation, and matrix deposition. Encapsulation in microspheres (e.g., BMP-2/VEGF in PLGA [54] or BMP-6 in alginate microspheres [55]) enhances osteogenesis, calcification, and cell proliferation. Surface adsorption (periostin on genipin–crosslinked CS [56]) increases osteocyte density, bone volume, and collagen fiber content, while also accelerating regeneration under unloaded conditions [57,58,59,60,61]. Immobilization on heparinized scaffolds (BMP-2 on Hep–CS [62,63]) prolongs factor release (up to 28 days) and significantly increases ALP activity, mineralization, and osteogenic gene expression [64,65,66,67,68,69,70,71,72,73]. Importantly, dual-factor systems such as BMP-2 combined with VEGF or PDGF have demonstrated synergistic effects, resulting in faster defect filling, greater bone volume, and more organized collagen deposition. The highest new bone area ratio (23.6%) was achieved with implanted scaffolds containing BMP-2 + VEGF. When the scaffold contained only BMP-2, this figure was 18.8% [74]. Thus, both the choice of bioactive agent and the method of incorporation can be adjusted to the specific requirements of the defect site and the desired regenerative timeline [75] (Table 1).

This systematic review aims to comprehensively evaluate strategies for application of chitosan-based scaffolds with growth factors for bone regeneration. Owing to its biocompatibility, biodegradability, and structural similarity to glycosaminoglycans, chitosan has attracted considerable interest as a comprehensive biomaterial in bone tissue engineering. Nevertheless, chitosan scaffolds often require functional enhancement through the incorporation of bioactive agents to address the complex biological and mechanical demands of bone repair [86]. Such functionalization improves osteoinductivity, osteoconductivity, and the overall regenerative performance of chitosan-based scaffolds [87]. Although numerous experimental studies describe various functionalization approaches, the literature lacks a systematic comparison of these methods, their mechanisms, and their quantitative effects on scaffold performance. A detailed assessment of factors influencing functionalization efficiency—including agent type, loading technique, release kinetics, and interactions with the chitosan matrix—is crucial for understanding their clinical potential and limitations. By consolidating and critically analyzing available evidence, this review aims to identify the most effective strategies and highlight existing knowledge gaps. This evidence-based synthesis may indicate the possibilities for design next-generation scaffolds and accelerate the translation of chitosan-based biomaterials into predictable and clinically effective solutions for bone regeneration.

## 2. Results

### 2.1. Study Selection

The initial database search across PubMed, Scopus, and Web of Science identified 367 potentially relevant records. After removal of duplicates, 226 unique articles remained for screening. Title and abstract screening led to the exclusion of 196 articles that did not investigate chitosan-based scaffolds functionalized with growth factors for bone regeneration. Of the 30 articles assessed in full text, 7 could not be retrieved, 1 was available only in Chinese, and 5 did not meet the predefined inclusion criteria. Ultimately, 17 studies fulfilled the eligibility requirements and were included in the qualitative synthesis. A meta-analysis was not feasible due to substantial heterogeneity among the included studies in terms of experimental design (animal models, defect sizes, follow-up duration), scaffold composition (crosslinking methods, composite materials), types and dosages of growth factors, as well as outcome measures (release kinetics, histological scoring systems, imaging modalities).

### 2.2. General Characteristics of the Included Studies

The most frequently investigated growth factor was BMP-2, reported in 10 studies [51,52,54,62,63,76,77,78,79,80], of which seven were conducted in vivo [51,54,76,77,78,79,80]. Four studies used rat models [54,76,79,80], while three employed rabbits [62,77,78]. Scaffold compositions varied considerably: hydroxyapatite was combined with chitosan in three studies [51,62,79], and heparin in two [62,63]. Other modifications included polyethylene glycol diacrylate [13], octacalcium phosphate–mineralized graphene [80], Aloe vera [82], and calcium phosphate salts (CPS) introduced via the ISISA process [78].

Incorporation methods for BMP-2 also differed. In heparin-containing scaffolds, BMP-2 was chemically immobilized [62,63]. Alternative strategies included microspheres [54], co-incorporation into chitosan gel [76], grafting of the P24 peptide derived from BMP-2 [51], covalent coupling [52], immobilization with CPS and ISISA processing [78], encapsulation in bovine serum albumin nanoparticles [80], and preloading into mesoporous hydroxyapatite nanoparticles (mHANPs) [79]. One study also investigated IGF-1 in combination with BMP-2 [77].

Two studies examined BMP-6 in vitro, delivered either via alginate microspheres [55] or PHBV submicron particles [81]. VEGF was studied in four investigations [54,82,83,84], three of which employed microsphere-based delivery [54,83,84]. Three of these were in vivo studies, two in rabbits [54,84] and one in guinea pigs (Cavia cobaya) [82]. De la Riva et al. [84] also explored PDGF by dissolving it in the aqueous phase of brushite and embedding VEGF in alginate microspheres. In vitro evaluation of bFGF in chitosan–hydroxyapatite scaffolds was reported by Tigli et al. [85], while Barakzai et al. [56] investigated periostin within genipin-crosslinked chitosan scaffolds in a rat model.

To enable comparative evaluation, many studies employed multiple outcome assessment methods. Computed tomography was reported in five studies [51,52,54,56,78], and X-rays in two [77,79]. Histological examination was the most common technique, performed in nine studies [51,54,56,76,77,78,79,80,84]. Eleven studies measured ALP activity [51,52,54,55,56,62,63,78,79,80,83], while calcium deposition was assessed in six [51,54,62,63,79,81]. ELISA testing was conducted in three studies [63,76,78]. In addition, Tigli et al. [85] uniquely analyzed fluorescence and release kinetics (Table 2).

### 2.3. Main Study Outcomes

#### 2.3.1. Histological and Imaging Outcomes

Histological and imaging analyses consistently demonstrated improved bone regeneration in functionalized scaffolds. Sanjaya et al. [76] observed a higher proportion of woven bone and more osteoblasts in rat calvarial defects treated with BMP-2 + chitosan compared to single-component groups. Nandi et al. [77] reported denser bone tissue, accelerated healing, and earlier appearance of Haversian systems in rabbit tibiae treated with IGF-1 or BMP-2. Wang et al. [54] achieved complete filling of mandibular bone defects in rabbits within 12 weeks using BMP-2 + VEGF in microspheres with ADSCs. Guzman et al. [78] demonstrated enhanced trabecular bone formation in rabbit tibiae with BMP-2 + CPS scaffolds processed by ISISA. Barakzai et al. [56] showed greater bone volume and collagen deposition in periostin-functionalized scaffolds under non-weight-bearing conditions. Xie et al. [80] and Qiu et al. [79] confirmed that BMP-2-functionalized chitosan composites significantly enhanced bone formation in rat cranial defects.

#### 2.3.2. Enzymatic Activity (ALP, OCN, PDGF, ELISA)

Multiple studies reported elevated enzymatic markers of osteogenesis. Sanjaya et al. [76] observed significantly higher osteocalcin (OCN) and PDGF levels in BMP-2 + chitosan scaffolds. Yun et al. [62,63] demonstrated that heparinized scaffolds prolonged BMP-2 release and significantly increased ALP activity, calcium deposition, and osteogenic gene expression. Chen et al. [51] confirmed that P24 peptide–grafted scaffolds enhanced ALP and mineralization in vitro and in vivo. Soriente et al. [52] reported higher ALP and OCN levels in BMP-2–functionalized CS-PEG scaffolds. Soran et al. [55] and Demirtaş et al. [81] also observed higher ALP activity and calcification in BMP-6–functionalized scaffolds, particularly when dual factors were used.

#### 2.3.3. Gene Expression of Osteogenic Markers

Several studies reported upregulation of osteogenic genes in functionalized scaffolds. Demirtaş et al. [81] observed increased expression of RunX2, Col1, OPN, and OCN with dual delivery of PDGF-BB and BMP-6. Liu et al. [83] showed that VEGF-functionalized HA/SA/CS scaffolds enhanced expression of ALP, BMP2, OPN, and RunX2. Qiu et al. [79] demonstrated upregulation of RunX2, ALP, Col1, and OCN in BMP-2–loaded silk fibroin–chitosan scaffolds. Yun [63] reported higher expression of osteocalcin and osteopontin in BMP-2/heparinized scaffolds compared to controls.

#### 2.3.4. Mineralization and Calcium Deposition

Functionalized scaffolds were consistently associated with enhanced mineralization. Soran et al. [55] observed greater calcification in BMP-6–loaded alginate microspheres compared to free BMP-6. Chen et al. [51], Soriente et al. [52], and Yun et al. [62] confirmed improved calcium deposition in BMP-2 and P24-functionalized scaffolds. Demirtaş et al. [81] reported early calcium deposits within 7 days in dual-loaded scaffolds, while Liu et al. [83] showed stronger Alizarin Red staining in VEGF-loaded scaffolds. Tigli et al. [85] indirectly linked hydroxyapatite addition to extended growth factor release and improved mineralization potential.

#### 2.3.5. Cell Proliferation and Morphology

Functionalized scaffolds enhanced cell adhesion, proliferation, and morphology. Sanjaya et al. [76] and Nandi et al. [77] reported higher osteoblast counts in BMP-2 and IGF-1 groups. Chen et al. [51] and Soriente et al. [52] observed increased BMSC and hMSC proliferation with P24 and BMP-2–modified scaffolds. Liu et al. [83] demonstrated greater MSC adhesion and proliferation in VEGF-functionalized scaffolds. Demirtaş et al. [81] confirmed improved spreading and cell–cell interactions in dual-factor scaffolds. Xie et al. [80] reported enhanced BMSC proliferation and osteogenic differentiation with BMP-2-loaded nanoparticles, while Qiu et al. [79] found increased BMSC attachment and matrix production. Guzman et al. [78] observed higher proliferation of C2C12 myoblasts in BMP-2 + CPS scaffolds. Barakzai et al. [56] additionally confirmed greater osteocyte density and viability in periostin-coated scaffolds.

#### 2.3.6. Angiogenesis and VEGF Expression

Angiogenesis was significantly improved in dual- and VEGF-containing systems. Wang et al. [54] showed that BMP-2 + VEGF synergistically promoted osteogenesis and angiogenesis, leading to rapid defect repair. De la Riva et al. [84] found that PDGF enhanced early bone formation, VEGF supported later-stage regeneration, and the combination yielded the largest trabecular area and multilayered osteoblasts. Sularsih et al. [82] demonstrated significantly higher VEGF expression and woven bone formation with chitosan–Aloe vera scaffolds. Nandi et al. [77] also observed intensified angiogenesis in GF-treated groups.

#### 2.3.7. Release Kinetics and Delivery Strategies

Several studies highlighted the importance of delivery strategies in prolonging growth factor activity. Yun et al. [62] demonstrated sustained BMP-2 release up to 28 days with heparinized scaffolds. Tigli et al. [85] showed that hydroxyapatite extended bFGF release from 10 to 20 h to 7 days. Chen et al. [51] reported stable release of the P24 peptide for 90 days. Guzman et al. [78] achieved controlled release of BMP-2 from CPS-modified scaffolds, enhancing long-term osteoinduction. Xie et al. [80] encapsulated BMP-2 in BSA nanoparticles, achieving sustained release and improved bioactivity. Qiu et al. [79] confirmed slower BMP-2 release and higher retention with mHANPs. De la Riva et al. [84] demonstrated a rapid, initial release of PDGF (approximately 45% in the first 24 h) and a more controlled release of VEGF (~13% in the first 24 h, ~64% within 3 weeks), which allowed for the sequential activation of proliferation and angiogenesis. In the study by Demirtaş et al. [81], the system provided almost complete release of PDGF-BB within 14 days (~90%) and a slower, partial release of BMP-6 (~50% in 14 days), reflecting the natural sequence of bone healing processes. Nandi et al. [77], on the other hand, did not perform classical in vitro studies, but described the gradual release of IGF-1 and BMP-2 in vivo as a result of enzymatic degradation of chitosan. In the work of Wang et al. [54], a rapid burst release within 10 days (66.8% BMP-2 and 65.2% VEGF) was reported, followed by a stable release phase up to 28 days, while maintaining the bioactivity of the released proteins (see Table 3).

### 2.4. Quality Assessment

For all of the nine questions, 14 papers received a positive answer to nine of them [51,52,54,55,56,62,63,77,78,79,81,82,83,85] and 3 papers received a positive answer to eight of them [76,80,84] (see Table 4).

### 2.5. Risk of Bias in Included Studies

The risk of bias for each included study was assessed using the Joanna Briggs Institute (JBI) checklist for quasi-experimental designs (non-randomized studies). Fourteen studies fulfilled all nine quality criteria, while three fulfilled eight. The most frequent limitations were the absence of long-term follow-up or incomplete reporting of blinding procedures. Despite these shortcomings, the overall methodological quality of the included studies was high, and the risk of bias was considered low to moderate. Table 4 provides a detailed summary of the assessment for each study.

### 2.6. Reporting Biases

We also evaluated the potential for reporting biases, particularly selective outcome reporting and publication bias. Although no clear evidence of selective reporting was identified in the included studies, the limited number of papers (n = 17) and the heterogeneity of outcome measures suggest that some degree of reporting bias may still be present. Furthermore, negative or inconclusive findings may be underrepresented in the available literature. As quantitative synthesis (meta-analysis) was not feasible, statistical tools such as funnel plots or Egger’s test could not be applied. To minimize such biases in future research, broader search strategies, inclusion of gray literature, and preregistration of study protocols are recommended.

## 3. Discussion

This systematic review aimed to comprehensively evaluate strategies for applying chitosan-based scaffolds with growth factors for bone regeneration. Among the 17 included studies, BMP-2 [88] was the most frequently investigated growth factor, while VEGF [54,82,83,84,89], BMP-6 [55,81,90], IGF-1 [77], PDGF [81,84], bFGF [85], periostin [56], and the BMP-2–derived P24 peptide [51] were also studied. Several works combined chitosan with additional scaffold components, including hydroxyapatite [51,54,79,91,92], heparin [62,63,93], polyethylene glycol diacrylate [52], graphene mineralized with octacalcium phosphate [80], genipin [56], Aloe vera [82,94], calcium phosphate salts [78], and silk fibroin [80]. Bone regeneration outcomes were assessed using computer tomography in five studies [51,52,54,56,78], X-ray imaging in two [77,79], and histological examination in nine [51,54,56,76,77,78,79,80,84]. Eleven studies measured ALP activity [51,52,54,55,56,62,63,78,79,80,83], and six reported calcium deposition [51,54,62,63,79,81]. ELISA testing was performed in three studies [63,76,78]. Overall, all studies confirmed that functionalization of chitosan with bioactive agents enhanced osteogenesis. The addition of BMP-2, VEGF, or IGF-1 accelerated bone formation and angiogenesis [54,76,77,82]. Increased osteocalcin levels, osteoblast counts, osteoinduction, and cell proliferation were consistently reported after incorporation of BMP-2, PDGF, VEGF, or BMP-6 [76,78,81,84,95]. Dual delivery of BMP-6 and PDGF-BB further upregulated RunX2, Col1, OPN, and OCN expression [81]. Scaffold modifications such as heparinization or hydroxyapatite addition enabled prolonged release of growth factors, sustaining their biological activity [3,8]. Importantly, the delivery strategy determined efficacy: BMP-6 encapsulated in alginate microspheres produced significantly greater osteogenesis than free BMP-6 [55].

The application of chitosan-based scaffolds combined with growth factors is considered one of the most promising strategies to enhance bone regeneration [96,97]. Numerous experimental studies have shown that such systems not only promote osteoproliferation but also significantly increase osteogenic gene expression (RunX2, Col1, OPN, OCN), ALP activity, and matrix mineralization [51,52,55,62,76,79,80,81,83,98]. The majority of findings suggest that chitosan scaffolds actively contribute to creating a biologically favorable microenvironment for bone repair [99,100]. This effect is achieved through the interaction between scaffold architecture, cellular adhesion, and the sustained release of growth factors [101,102]. The combination of these factors results in enhanced cell proliferation and more efficient osteoblast differentiation [84,103]. The ability to stimulate early-stage cell activity appears particularly important in accelerating the healing of bone defects and may ultimately reduce the time required for functional recovery in clinical practice [104]. In this context, chitosan-based composites demonstrate potential not only as osteoconductive scaffolds but also as bioactive materials capable of supporting the multiple mechanisms involved in bone repair [105,106].

In the reviewed studies, the density and quality of newly formed bone tissue were consistently enhanced when growth factors were incorporated into chitosan scaffolds. Particularly notable were dual growth factor systems, such as PDGF-BB + BMP-6 [81]. PDGF-BB strongly increased osteoblast proliferation, BMP-6 promoted differentiation, while their combination induced a synergistic effect, particularly visible in mineralization and osteocalcin expression. Similarly, Wang et al. [54] reported that after 12 weeks, the BMP-2 and BMP-2/VEGF groups achieved more than 86% new bone, but only the combination produced tissue density (CT ~1623 HU) close to normal and improved lamellar remodeling, while VEGF alone had a moderate effect (12.9% NB). De la Riva et al. [84] showed a significant increase in the new bone area in the PDGF group (0.82 mm^2^) and especially in the PDGF + VEGF group (1.74 mm^2^) compared to the control (0.12 mm^2^), which corresponded to an improvement of up to 14-fold. Sularsih et al. [82] further confirmed that VEGF-functionalized scaffolds promoted both neovascularization and dense bone fibrosis. Collectively, these outcomes indicate that scaffold modifications not only stimulate osteogenesis but also promote angiogenesis, thereby ensuring adequate vascularization and nutrient supply for developing bone. This dual action supports the transition from immature woven bone to compact, functionally stable structures [106,107]. The observed synergy suggests that carefully designed scaffold systems can provide superior regenerative outcomes, particularly in large or complex defects where spontaneous repair is insufficient. Moreover, the simultaneous stimulation of osteogenesis and angiogenesis may play a decisive role in reducing the risk of non-union and improving the long-term biomechanical performance of regenerated bone [108,109].

Another vital aspect of scaffold performance is the sustained and controlled release of growth factors, which has been extensively investigated in experimental studies [110,111,112]. Scaffold modifications such as the incorporation of hydroxyapatite, heparin, or immobilized calcium phosphate salts have been shown to prolong release kinetics from only a few hours to several days or even weeks [62,78,85]. Similarly, encapsulation strategies using microspheres or nanoparticles (e.g., BMP-2 or VEGF in PLGA microspheres [54,84,113], BMP-6 in alginate microspheres [55], or BMP-6 in PHBV submicron particles [81]) provided gradual and extended release profiles, maintaining factor bioactivity over time. This prolonged availability ensures that osteogenic stimulation persists not only in the early stages of healing following implantation but also during later phases of bone maturation [114,115]. A continuous supply of bioactive agents supports cell proliferation, differentiation, and extracellular matrix deposition. When combined with dual delivery systems [54,77,81,84], controlled release further enhances both osteogenesis and angiogenesis, producing superior regenerative outcomes. From a clinical perspective, scaffold systems with controlled and sustained release may offer a more effective and predictable approach to bone defect repair by ensuring long-term biological activity, enhanced tissue integration, and improved biomechanical stability of regenerated bone. Importantly, the capacity to modulate release kinetics provides opportunities to tailor scaffold design to specific clinical scenarios, ranging from small defects to critical-sized bone injuries.

This systematic review has several limitations that should be acknowledged. First, the number of eligible studies was relatively small (n = 17), and most were preclinical investigations conducted in rodents or rabbits, which restricts the direct translation of findings to humans. Considerable heterogeneity was observed in scaffold composition, methods of growth factor incorporation, dosing strategies, and outcome measurements, which precluded the possibility of conducting a meta-analysis. Moreover, many studies had short follow-up periods, limiting insights into long-term scaffold performance and potential adverse effects, such as ectopic bone formation or uncontrolled release of bioactive agents. Publication bias cannot be ruled out, as negative or inconclusive results may be underreported. Future research should focus on standardized experimental protocols that would allow for meaningful comparisons across studies, including harmonized dosing strategies, release kinetics assessment, and outcome measures. Large-animal models that better replicate the biomechanical and biological environment of human bone defects are needed before clinical translation. Clinical trials will be necessary to validate both the safety and efficacy of functionalized chitosan scaffolds, with particular emphasis on critical-sized defects and load-bearing sites. Furthermore, advanced scaffold designs combining multiple growth factors with immunomodulatory strategies or stem/progenitor cell delivery may offer synergistic effects and should be explored [116]. Ultimately, translational studies integrating bioengineering, molecular biology, and clinical expertise will be essential to optimize scaffold performance and accelerate their application in routine clinical practice.

## 4. Materials and Methods

### 4.1. Focused Question:

This systematic review was designed according to the PICO framework [117]:

Population (P): experimental models using chitosan-based scaffolds for bone regeneration,

Intervention (I): functionalization of chitosan scaffolds with growth factors,

Comparator (C): non-functionalized chitosan scaffolds (without growth factors),

Outcome (O): enhancement of bone regeneration, assessed by histological, radiological, biochemical, or molecular parameters.

The focused review question was: does the incorporation of growth factors into chitosan-based scaffolds improve bone regeneration compared with non-functionalized chitosan scaffolds?

### 4.2. Protocol

**Figure 3:**
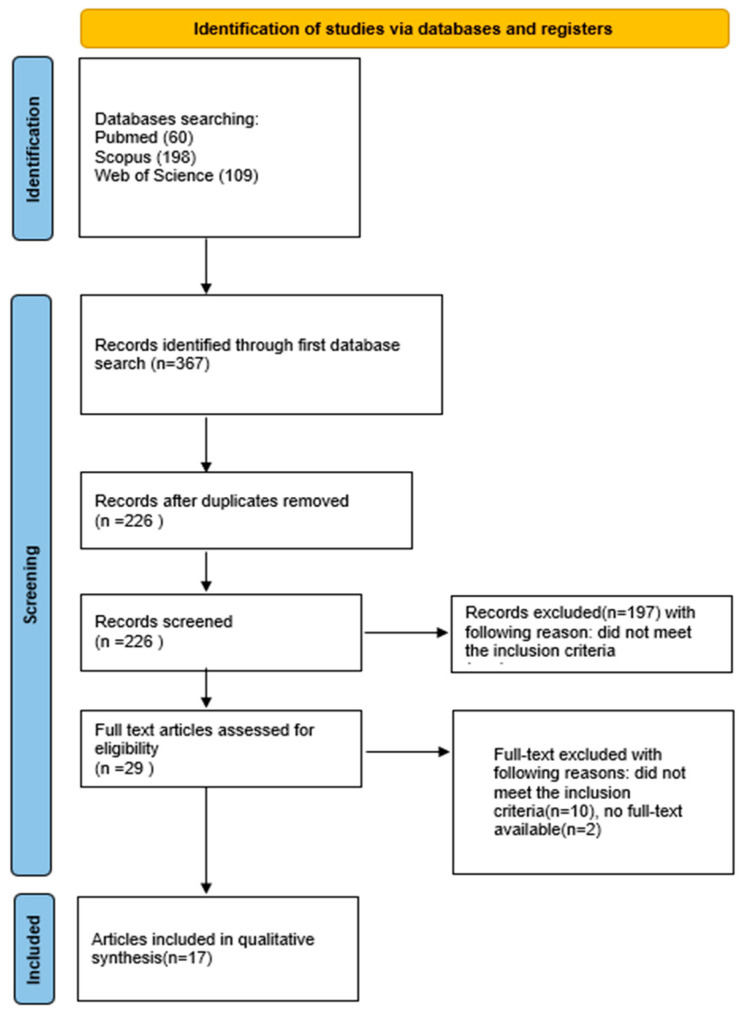
PRISMA 2020 flow diagram.

The selection process for articles in the systematic review was carefully outlined following the PRISMA flow diagram (Figure 3) [118]. The systematic review was registered on the Open Science Framework under the following link: https://osf.io/zhdm8 (accessed on 23 August 2025).

### 4.3. Eligibility Criteria

Studies were considered acceptable for inclusion in the review if they met the following criteria [119,120,121,122,123,124,125,126,127]:

Inclusion criteria:

- Studies examining the effect of adding growth factors to chitosan scaffolds on bone regeneration.
- In vitro studies
- In vivo studies
- Studies with a control group;
- Studies in English;
- Non-randomized controlled clinical trials (NRS); and
- Randomized controlled clinical trials (RCT).

The exclusion criteria the reviewers agreed upon were as follows:

Studies not examining the effect of adding growth factors to chitosan scaffolds on bone regeneration [119,120,121,122,123,124,125,126,127].

- Non-English papers;
- Clinical reports;
- Opinions;
- Editorial papers;
- Review articles;
- No full-text accessible; or
- Duplicated publications.

No restrictions were applied with regard to the year of publication.

### 4.4. Information Sources, Search Strategy, and Study Selection

A comprehensive literature search was conducted in July 2025 across three electronic databases: PubMed, Scopus, and Web of Science (WoS). The search aimed to identify studies investigating the effect of growth factor incorporation into chitosan-based scaffolds for bone regeneration. Search terms combined the scaffold descriptor with individual growth factors and were applied to titles and abstracts only. The complete search strategies for each database are provided below:

PubMed: (“chitosan scaffold”[Title/Abstract]) AND (“growth factor”[Title/Abstract] OR “BMP-2”[Title/Abstract] OR “VEGF”[Title/Abstract] OR “FGF”[Title/Abstract] OR “TGF-beta”[Title/Abstract] OR “periostin”[Title/Abstract] OR “PDGF”[Title/Abstract] OR “IGF-1”[Title/Abstract] OR “EGF”[Title/Abstract] OR “ANG-1”[Title/Abstract] OR “ANG-2”[Title/Abstract] OR “GDF-5”[Title/Abstract] OR “SDF-1”[Title/Abstract] OR “osteopontin”[Title/Abstract])

Web of Science (WoS): TS = (“chitosan scaffold”) AND TS = (“growth factor” OR “BMP-2” OR “VEGF” OR “FGF” OR “TGF-beta” OR “periostin” OR “PDGF” OR “IGF-1” OR “EGF” OR “ANG-1” OR “ANG-2” OR “GDF-5” OR “SDF-1” OR “osteopontin”)

Scopus: TITLE-ABS-KEY(“chitosan scaffold”) AND TITLE-ABS-KEY(“growth factor” OR “BMP-2” OR “VEGF” OR “FGF” OR “TGF-beta” OR “periostin” OR “PDGF” OR “IGF-1” OR “EGF” OR “ANG-1” OR “ANG-2” OR “GDF-5” OR “SDF-1” OR “osteopontin”)

Following the database searches, all retrieved records were imported into a reference management system, and duplicates were removed. Two reviewers independently screened the remaining articles by title and abstract to exclude studies not meeting the inclusion criteria. Full-text articles were subsequently assessed for eligibility, with only those meeting all predefined criteria and available in full text included in the final qualitative synthesis.

### 4.5. Data Collection Process and, Data Items

Six reviewers (M.L., Z.N., J.K., S.K., M.M.) independently evaluated the identified publications to determine whether they fulfilled the inclusion requirements. From each eligible article, the following data were collected: name of the first author, year of publication, study design, article title, type of growth factor applied, and its reported influence on bone regeneration. All extracted information was organized and stored in a standardized Excel spreadsheet.

### 4.6. Risk of Bias and Quality Assessment

During the first stage of selection, titles and abstracts were independently assessed by all reviewers to reduce potential bias. Two blinded reviewers (J.M. and M.D.) independently evaluated the methodological quality of each included study using the Joanna Briggs Institute (JBI) checklist for quasi-experimental designs (non-randomized studies). This tool consists of nine targeted questions designed to assess study validity and reliability. In case of disagreement, issues were first discussed collectively, and inter-rater reliability was objectively quantified using Cohen’s kappa coefficient [128]. This ensured that the assessment process was both transparent and reproducible, and reduced the risk of subjective bias.

### 4.7. Quality Assessment

Two blinded reviewers (J.M. and M.D.) independently evaluated the methodological quality of each included study using the Joanna Briggs Institute (JBI) checklist for quasi-experimental designs (non-randomized studies) [129]. This assessment tool comprises nine targeted questions enables a reliable quality assessment, is methodologically recognized, enhances transparency and repeatability, and is consistent with international standards.

- Is it clear in the study what is the “cause” and what is the “effect”?
- Were the participants included in any similar comparisons?
- Were the participants included in any comparisons receiving similar treatment/care, other than the exposure or intervention of interest?
- Was there a control group?
- Were there multiple measurements of the outcome both before and after the intervention/exposure?
- Was a follow up completed, and if not, were the differences between groups in terms of their follow up adequately described and analyzed?
- Were the outcomes of the participants included in any comparisons measured in the same way?
- Were the outcomes measured in a reliable way?
- Was an appropriate statistical analysis used?

Each criterion on the checklist was rated as “yes,” “no,” “unclear,” or “not applicable.” Whenever reviewers assigned different responses, the inconsistencies were discussed until a consensus was reached. Inter-rater reliability was assessed using Cohen’s kappa, computed with MedCalc software (version 23.1.7; MedCalc Software Ltd., Brussels, Belgium). The resulting kappa value was 0.91 (*p* < 0.001), which corresponds to a high level of concordance and indicates almost perfect agreement among the reviewers.

## 5. Conclusions

This review demonstrates that the incorporation of growth factors into chitosan-based scaffolds significantly enhances bone regeneration compared with chitosan alone. Among them, BMP-2 remains the most extensively validated osteogenic mediator, consistently promoting osteoblast differentiation and mineralized matrix formation. Other factors, including BMP-6 and IGF-1, provided additional osteogenic stimulation through increased cell proliferation and metabolic activity, while VEGF primarily supported neovascularization. PDGF and bFGF contributed to extracellular matrix remodeling and fibroblast expansion, and periostin further enhanced collagen deposition and bone volume. Importantly, controlled-release strategies such as microsphere encapsulation, heparinization, and mineral-based binding prolonged the bioactivity of incorporated molecules, leading to more stable and predictable regenerative outcomes. Dual-factor systems, such as BMP-2 with VEGF or PDGF-BB with BMP-6, demonstrated synergistic effects by simultaneously stimulating osteogenesis and angiogenesis, thereby accelerating defect healing.

Altogether, the available evidence highlights functionalized chitosan–growth factor scaffolds as a promising bioactive material capable of improving both the quality and speed of bone healing. However, given the heterogeneity of study designs and the predominance of preclinical models, further standardized research and clinical trials are needed to validate their long-term safety, efficacy, and translational potential in the treatment of large and complex bone defects.

## Figures and Tables

**Table 1:** Bioactive factors used in chitosan-based scaffolds, their biological role, and functionalization strategies.

Bioactive Factor	Main Biological Role	Functionalization Strategy (Chemical/Physical)	References
BMP-2	Potent osteoinductive factor; promotes differentiation of mesenchymal stem cells into osteoblasts	Grafting (P24 peptide), covalent coupling, microsphere encapsulation, nanoparticle incorporation, heparinization	[51,52,54,62,63,76,77,78,79,80]
BMP-6	Strong osteogenic factor; stimulates osteoblast activity and matrix mineralization	Encapsulation in alginate microspheres or PHBV submicron particles	[55,81]
IGF-1	Stimulates proliferation and differentiation of osteoblasts; enhances collagen synthesis	Physical adsorption, soaking into CS scaffolds	[77]
VEGF	Stimulates angiogenesis and vascularization within regenerating tissue	Encapsulation in alginate or PLGA microspheres, composite reinforcement (e.g., Aloe vera, HA-CS)	[54,82,83,84]
bFGF (FGF-2)	Enhances proliferation of mesenchymal stem cells and angiogenesis; delays differentiation	Embedding in CS–HA scaffolds, release control via HA interactions	[85]
PDGF	Promotes proliferation and angiogenesis; recruits progenitor cells	Combined encapsulation (alginate microspheres, brushite–CS scaffolds)	[81,84]
Periostin	Extracellular matrix protein; supports adhesion, migration, and mechanical stress adaptation	Physical adsorption on genipin–crosslinked chitosan	[56]

**Table 2:** General characteristics of the included studies.

Study	Aim of the Study	Material and Methods	Results	Conclusions
Wang et al. [54]	To determine whether an integrated, injectable hydrogel platform delivering BMP-2, VEGF, and adipose-derived mesenchymal cells (ADSCs) supports the regeneration of vascularized bone tissue in rabbit mandibular defects.	Chitosan hydrogel with PLGA/nHA microspheres containing BMP-2 and VEGF was prepared and implanted together with ADSCs into a critical bone defect in the mandible of rabbits; bone lesion formation was assessed by computed tomography and macro- and histological observations for 12 weeks	Hydrogel with BMP-2 and VEGF + ADSC significantly accelerated bone formation, shortened healing time, and improved callus remodeling compared to the control and VEGF-alone groups, with full defect filling after 12 weeks	Co-delivery of VEGF and BMP-2 along with ADSCs through chitosan hydrogel effectively promotes simultaneous osteogenesis and angiogenesis, making this strategy very promising for regenerative bone engineering.
Yun et al. [62]	We investigated whether heparin-coated chitosan scaffolds capable of sustained release of BMP-2 improved osteoblast function compared to traditional chitosan or BMP-2/chitosan.	In vitro, the osteoblast cell line MG-63 cultured on three types of scaffolds (chitosan, BMP-2/chitosan, and BMP-2/heparin/chitosan) was used. Proliferation, ALP activity, calcium deposition, and expression of osteogenic genes were assessed.	BMP-2/heparin/chitosan scaffolds provided a more continuous BMP-2 efflux, which resulted in a significant increase in ALP activity and calcium deposition in osteoblast cells compared to the other groups	The heparin coating of the chitosan scaffold enables sustained release of BMP-2, which positively influences osteoblast function—a promising strategy for bone regeneration.
Sanjaya et al. [76]	To check whether the use of a combination of rhBMP-2 and a chitosan scaffold accelerates the regeneration of calvarial defects in Wistar rats.	Twenty-four male Wistar rats were used, randomly assigned to 4 groups (control, rhBMP-2 only, chitosan scaffold only, rhBMP-2 + chitosan combination), and healing was assessed after 4 weeks by measuring osteocalcin expression, osteoblast number, fibrous bone area, and PDGF level.	The combination of rhBMP-2 and chitosan scaffold significantly increased osteocalcin expression, osteoblast number, fibrous bone area, and PDGF compared to single interventions and the control group	The combination of rhBMP-2 with chitosan scaffold effectively accelerates bone regeneration in skull defects in rats, exceeding the effects of rhBMP-2 or chitosan alone
Tigli et al. [85]	To assess whether chitosan scaffolds containing dexamethasone or bFGF with hydroxyapatite can provide controlled, sustained drug release for tissue engineering purposes.	Pure chitosan or chitosan-HA scaffolds were prepared by incorporating different doses of dexamethasone (300–900 ng) or bFGF (50–100 ng) per dry sample (3 mg) and analyzing the release in DPBS buffer in vitro for ≤5 days using UV-VIS spectrophotometry.	The scaffolds demonstrated continuous release of dexamethasone for approximately 5 days, while bFGF was completely released within 10–20 h. The addition of hydroxyapatite allowed for prolonged bFGF release to up to 7 days due to electrostatic interactions with HA.	The chitosan-HA design enables controlled and sustained release of both dexamethasone and bFGF, making these materials a promising solution for applications in bone and periodontal tissue regeneration.
Liu et al. [83]	Development and testing of a novel composite scaffold of hydroxyapatite, sodium alginate and chitosan capable of simultaneously releasing vancomycin and VEGF to accelerate bone regeneration and prevent infections.	The scaffolds were encapsulated within a VEGF microsphere with a vancomycin-containing layer and their physical properties, drug release rate, in vitro bactericidal effect against S. aureus, and osteogenic potential of MSCs were tested.	The HA_6_(SA/CS)_4_@VAN/VEGF scaffold exhibited optimal porosity, controlled degradation, dual drug release within the designed time frame, potent antibacterial activity against S. aureus, good biocompatibility, and support for MSC proliferation and osteogenesis	The HA-SA-CS composite scaffold with embedded VEGF and vancomycin shows promising potential as a bone tissue engineering material, combining mechanical, antibacterial, and pro-osteogenic properties.
Barakzai et al. [56]	To investigate whether chitosan implants containing periostin accelerate bone regeneration in the absence of mechanical loading.	The study involved 36 rats divided into three groups (control, chitosan, and chitosan with periostin). Scaffolds were implanted into femoral defects, and regeneration was assessed histologically and morphometrically after 4 and 8 weeks.	The periostin group showed significantly greater bone regeneration, better tissue organization, and increased expression of osteogenesis markers compared to the other groups.	The addition of periostin to a chitosan scaffold effectively promotes bone healing under non-weight-bearing conditions, suggesting the clinical potential of this combination in the treatment of bone defects.
Chen [51]	Investigate whether the CS-P24/HA scaffold, which delivers the P24 peptide from BMP2, can be used in bone regeneration procedures.	P24 and BMP were incorporated into the chitosan structure. HA was then added to form the CS-5%P24/HA or CS-10%P24/HA scaffold. CS-HA was used as a control. In vitro, the release of P24 from the samples into a PBS solution was assessed chromatographically over 90 days. Quantitative assessment by HPLC. Rat bone marrow stem cells were seeded onto the CS-5%P24/HA and CS-10%P24/HA scaffolds. Cell proliferation, ALP activity, Ca deposition and osteogenic mRNA levels were assessed. In vivo: Three groups of rats were implanted with CS/HA, CS-5%P24/HA and CS-10%P24/HA. After sacrifice, scaffold samples were analyzed by microtomography. Assessment: BMC, BMD, TMD, histology and immunohistochemistry (expression of: Ocn, Nestin and CD31. In vivo no. The following were implanted into the skull defects of rats: CS/HA, CS-5%P24/HA and CS-10%P24/HA. After sacrifice, the study was conducted as in No. 1.	In vitro: linear and stable release of P24 from both test samples over 90 days. In vivo: stem cell count, alkaline phosphatase (ALP) activity, cell proliferation, mRNA levels, calcium (Ca) levels, quantity and quality of regenerated bone were all higher with CSP24/HA than with CS/HA.	P24-containing chitosan scaffolds promote faster and better bone regeneration.
Soriente [52]	The creation of a bioactive chitosan scaffold that enhances osteogenesis.	Two types of bioactive chitosan scaffold were created: 1) with BMP (organic), and 2) with HA nuclei (inorganic). The scaffold structure was assessed using SEM and MicroCT. In vitro: Peptide release from the BMP scaffolds was analyzed by chromatography. Cell proliferation on the scaffolds was evaluated using the Alamar blue assay. ALP activity was assessed on hMSC scaffolds. Quantitative OCN measurements from the substrate were used as a marker of osteogenesis.	Higher ALP and OCN values were observed for the tested scaffolds containing BMP and biomineralized HA. The higher the concentration of BMP2, the better the cell morphology, i.e., the structure of osteoblasts. At lower concentrations, the morphology resembles that of fibroblasts. Faster cell proliferation was observed in the presence of BMP2.	Adding BMP2 and HA to chitosan scaffolds may encourage bone regeneration.
Soran [55]	The effect of adding alginate microspheres containing BMP-6 to chitosan gels on periodontal tissue regeneration.	Chitosan scaffolds containing alginate microspheres were prepared (1. empty; 2. enriched with BMP-6). In vitro: BMP-6 release measurement was performed using a fluorescence spectrophotometer. rBM-MSC cells were injected into: 1. CS, 2. CS + BMP-6, and 3. CS + BMP-6 + alginate. ALP and mineralization were analyzed using van Kossy, and cell morphology was assessed using SEM.	The most intense stem cell proliferation was observed in the BMP-6 plus microspheres group. The ALP level was highest in the BMP-6 plus microspheres group, significantly higher than in the free BMP-6 group. Calcification was also highest in this group. The BMP-6 plus microspheres group also exhibited the most complex stem cell morphology.	Chitosan scaffolds containing alginate microspheres and BMP-6 demonstrated the most effective delivery of factors that promote stem cell development.
Nandi [77]	How does the addition of IGF-1 and BMP-2 to a chitosan scaffold affect bone healing	Chitosan scaffolds were impregnated with IGF-1 and BMP-1. In vivo: rabbit tibiae with defects were filled with the following: A. no treatment, B. chitosan + IGF-1, or C. chitosan + BMP-1. Histopathological analysis was performed after 30 and 90 days following implant placement. Bone sections were analyzed under an Orthoplan microscope. The sections were also examined using SEM.	Compared to group A, there was greater osteoblast activity, a greater number of cells, and increased proliferation of bone tissue and vessels in groups B and C. Bone formation density was also significantly higher in groups B and C. Fluorescence in the microscopic image was much more intense in groups B and C.	The addition of IGF-1 and BMP-1 to chitosan significantly accelerates the regeneration and reconstruction of bone tissue.
Sularsih [82]	Analysis of the pores-size of chitosan-Aloe vera scaffold on alveolar bone healing and VEGF expressions after tooth removal (study was conducted on Cavia cobaya).	The study was conducted on 36 males of Cavia cobaya, divided into 6 groups (*n* = 6): negative control (without scaffolds), positive control (chitosan scaffold), treatment groups (chitosan–aloe vera scaffold). Data analyzed: Woven alveolar bone areas and VEGF expressions (by histopathological examination, ANOVA, LSD) Scaffold pore size (by SEM)	The largest woven alveolar bone and the highest expression on VEGF was observed in the treatment groups after 7 and 14 days (statistically significant difference between control and treatment group). Open pore interconnectivity was observed in chitosan- Aloe vera scaffolds.	Usage of chitosan–aloe vera scaffold (by its pore characteristics) increased VEGF expressions and woven alveolar bone areas.
Yun [63]	Development of novel bone-grafting scaffolds (BMP-2-immobilizingheparinized-chitosan scaffolds) And assessment of its osteogenic differentiation activity.	Chitosan scaffolds were functionalized with heparin and BMP-2 to enhance osteogenic potential. Measured factors: - Cell culture and proliferation: MG-63 osteosarcoma cells were cultured on scaffolds, cell proliferation was measured (CCK-8 assay) - Alkaline Phosphatase (ALP) Activity (assessed to confirm early osteoblast differentiation) - Calcium deposition—measured after 21 days (indication of osteogenic differentiation) - mRNA levels of osteocalcin and osteopontin—assessment of osteoblast differentiation (RT-PCR)	The results showed that BMP2-immobilizing heparinized-chitosan (BMP-2/Hep-chitosan) scaffolds significantly enhanced ALP activity and calcium deposition of the osteoblast cells when compared with chitosan scaffolds only. Also, mRNA expressions of osteocalcin and osteopontin of osteoblast cells cultured on BMP-2 (100 ng)/Hep-chitosan scaffolds were increased compared to chitosan scaffolds.	BMP-2/Hep-chitosan systems can increase osteoblast activity, therefore such systems are a platform to develop next generation transplant materials.
Guzman [78]	Evaluation of osteoinductive capability of the immobilized bone morphogenetic protein adsorbed to chitosan scaffold after manufacturing process.	Materials: chitosan (CHI), hydroxyapatite (HAp), urease, urea, and rhBMP-2. Hydrogel Preparation: CHI hydrogels were mixed with urea, urease, and optionally rhBMP-2 or CPS, then gelled at 37 °C for 24 h. ISISA Processing: Hydrogels were frozen, freeze-dried, and cryo-fractured into 2.5 mm scaffolds. In Vitro Assays: - Cell Proliferation and Viability: Alamar Blue and Calcein AM assays on C2C12 cells. - BMP-2 Activity: ALP activity was measured. - BMP-2 Release: ELISA at multiple time points. - In Vivo Assays: - Surgery: Tibial defect implantation in rabbits. - Micro-CT and Histology: Bone formation and histomorphometry analysis.	The results showed that scaffolds containing both rhBMP-2 and CPS-CHI enhanced bone regeneration, with rhBMP2-CPS-CHI scaffolds inducing significantly more new bone formation compared to the other scaffolds. While rhBMP2-CHI alone did not lead to increased bone growth, the combination of CPS with rhBMP-2 promoted a more effective release of the growth factor, resulting in superior osteoinductive effects. Histological and microCT analyses confirmed the presence of trabecular bone and chondral zones in defects treated with rhBMP2-CPS-CHI.	rhBMP2 was released in a controlled way from scaffold and retained its osteoinductive character after release. The multicomponent scaffold showed better regenerative abilities than the scaffolds containing only one of the components (CPS or rhBMP2 separately).
De la Riva [84]	Evaluation the effectiveness of a brushite- chitosan scaffold loaded with PDGF or a combination of PDGF and VEGF in supporting bone regeneration.	Brushite–chitosan scaffolds were developed, with PDGF combined in the cement liquid phase and VEGF encapsulated in alginate microspheres embedded in chitosan sponges. In vitro and in vivo release kinetics were examined using radiolabeled growth factors (^125^I). Implants were inserted into rabbit femur with bone defect and analyzed for growth factor release, distribution and bone regeneration.	Both in the in vivo and in vitro study PDGF was released faster than VEGF. Both factors stayed localized. Bone regeneration was enhanced in scaffolds containing PDGF and even more in those containing both—PDGF and VEGF.	PDGF supports early stages of healing, while VEGF delivery aids in later bone formation and vascularization. Bone regeneration after dual delivery of GF (PDGF + VEGF) was higher than single GF application. The brushite-chitosan scaffold allows localized, time- controlled release, supporting tissue regeneration.
Demirtaş [81]	This study aimed was to evaluate the positive effects of combined delivery of two growth factors—PDGF-BB (a mitogenic factor) and BMP-6 (an osteogenic factor)—loaded within a chitosan-based scaffold, on growth and maturation of pre-osteoblastic MC3T3-E1 cells.	PDGF-BB was loaded into gelatin microparticles and BMP-6 into PHBV submicron particles. These were then incorporated into 3D porous chitosan scaffolds prepared by freeze-drying. Different degradation rates led to faster PDGF-BB and slower BMP-6 release. MC3T3-E1 cells were seeded onto the scaffolds and analyzed over 21 days for viability, proliferation (MTT), morphology (SEM), and gene expression (RT-PCR).	Chitosan scaffolds loaded with both PDGF-BB and BMP-6 promoted greater cell proliferation and significantly enhanced expression of osteogenic markers (RunX2, Col1, OPN, OCN) compared to single-factor scaffolds. PDGF-BB was released rapidly, enhancing early cell proliferation, while BMP-6 had a sustained release supporting differentiation.	Chitosan serves as a biocompatible scaffold but needs incorporation of growth factors to promote osteogenesis effectively. The dual- release system using PDGF-BB and BMP-6 in chitosan scaffolds showed more effective than single-factor systems. This approach supports physiological mechanism of tissue repair.
Xie [80]	The aim of the study was to develop a biomimetic scaffold based on chitosan and graphene oxide, mineralized with octacalcium phosphate (OCP), and functionalized with bone morphogenetic protein-2 (BMP-2) and silver nanoparticles (Ag-NPs). The research focused on evaluating whether incorporating BMP-2 into the scaffold would improve its osteoinductive capacity and promoting bone tissue regeneration.	Porous scaffolds composed of GO and chitosan were fabricated and then mineralized using a supersaturated calcium phosphate solution to deposit OCP. BMP-2 was encapsulated in BSA nanoparticles stabilized with chitosan and immobilized on the scaffold via adsorption. Characterization included structural analysis, BMP-2 release studies, in vitro tests with bone marrow stromal cells (BMSCs), and in vivo implantation in rat cranial defect models.	Scaffolds with BMP-2-loaded nanoparticles exhibited sustained release of the growth factor, significantly enhancing BMSC proliferation and osteogenic differentiation compared to controls. In vivo, these scaffolds promoted more extensive new bone formation. The delivery of BMP-2 from nanoparticles was more effective than direct surface adsorption.	Incorporating BMP-2 into OCP-GO/CS scaffolds via nanoparticle encapsulation notably improved their osteoinductive properties. The controlled release of BMP-2 created a favorable microenvironment for bone regeneration.
Qiu [79]	The study aimed to develop a biomaterial-based scaffold that enables controlled and sustained release of—BMP-2, enhancing its biological effectiveness in bone regeneration.	Three types of scaffolds were prepared: one without BMP-2 (SCH), one with BMP-2 simply adsorbed on the surface (SCH-D), and one where BMP-2 was preloaded into mHANPs and then embedded in the scaffold (SCH-L). All were assessed through in vitro tests (release kinetics, ALP activity, gene expression) and in vivo in rat skull defects.	SCH-L showed a significantly slower initial BMP-2 release and higher retention of its bioactivity than SCH-D. In cell studies, SCH-L led to significantly higher ALP activity and gene expression (RunX2, Col I, ALP, OC). In vivo, SCH-L generated more uniform and extensive new bone formation.	Preloading BMP-2 into mHANPs resulted in a more effective and bioactive release profile. The SCH-L scaffold promoted stronger in vitro and in vivo bone regeneration.

**Table 3:** Detailed characteristics of included studies.

Authors	Study Model	Scaffold Composition	Bioactive Agent and Functionalization Strategy	Bone Regeneration Assessment Methods	Observed Effects on Bone Regeneration
Wang et al. [54]	In vivo, rabbit	Chitosan (CS) + nanohydroxyapatite (nHA) + PLGA microspheres	BMP-2/VEGF-loaded PLGA microspheres	3D-CT, histology (Masson’s stain), ALP, Ca deposition, in vitro bioactivity assessment	- VEGF: limited angiogenesis and regeneration - BMP-2: significant osteogenesis - BMP-2 + VEGF: synergistic angiogenesis + osteogenesis, complete defect filling after 12 weeks
Yun et al. [62]	In vitro—MG-63 cell line (osteoblasts)	CS + heparin (coating)	Chemically immobilized BMP-2 on a heparinized scaffold surface	ALP, Ca deposition, gene expression (OCN, OPN), cell proliferation (CCK-8)	- Extended BMP-2 release (up to 28 days) - Significantly higher ALP activity, mineralization, and osteogenic gene expression compared to the heparin-free scaffold
Sanjaya et al. [76]	In vivo, rat	Chitosan gel 3% in HPMC 5%	Co-administration/co-incorporation rhBMP-2	Histology (H&E)—osteoblast count and % woven bone tissue; ELISA: osteocalcin (OCN) and PDGF	significantly higher values of OCN, PDGF, % woven bone and osteoblast number compared to the control group
Tigli et al. [85]	In vitro	CS + HA	bFGF embedding/solvent sorption method	Dexamethasone and bFGF release study, fluorescence, UV, release kinetics	without HA—bFGF release in 10–20 h; with HA—extended to 7 days due to electrostatic interactions with HA
Liu et al. [83]	In vitro, rat	HA/sodium alginate (SA)/CS	Multilayer microspheres with VEGF inside	Adhesion (confocal), ALP (staining + semi-quantitative), mineralization (Alizarin Red), gene expression (qPCR: ALP, BMP2, OPN, RunX2); VEGF/VAN release; antibacterial tests (OD and inhibition zones)	greater cell adhesion, higher ALP activity, stronger mineralization and higher ALP/BMP2/OPN/RunX2 expression compared to the control group
Barakzai et al. [56]	In vivo, rat	Chitosan (2% *w*/*v*) cross-linked with genipin	Periostin Surface deposition/adsorption	Micro-CT, histology (H&E, toluidine, Masson’s trichrome), ALP activity	Higher osteocyte count, higher bone volume and collagen fiber percentage compared to the control group, good cell viability and degradation in vitro. Periostin accelerated regeneration under unloaded conditions.
Chen [51]	In vitro, rat BMSC In vivo, rat	CS-HA	P24 from BMP-2, chemical grafting on chitosan scaffolds	- ALP - calcium deposition - Micro-CT - histological examination - Immunohistochemistry - PCR	Scaffolds with P24 comparing to control group (no P24): - Greater cell adhesion, density and proliferation - A greater number of BMSCs - Increased mineralization and ALP. - Higher mRNA levels - More advanced osteogenesis -More advanced BMSC structure.
Soriente [52]	In vitro, human stem cell line	-CS + polyethylene gly- col diacrylate (20%) -CS + polyethylene gly- col diacrylate (40%)	BMP-2, covalent coupling on scaffold surface	- Micro CT - Alamar blue assay - ALP - OCN	Scaffold with BMP-2 comparing to biomineralized scaffolds: - Greater adhesion and proliferation of cells - Higher values of ALP and OCN.
Soran [55]	In vitro, rat stem cells	-CS + alginate microspheres	BMP-6; BMP-6 in alginate microspheres mixed with CS	- von - Kossa analysis(mineralization) - ALP - SEM	Group with alginate microspheres: - significantly greater osteogenesis in the microsphere group - much greater calcification - highest cells proliferation of all groups
Nandi [77]	In vivo, rabbit	Chitosan-based scaffold	IGF-1; BMP-2; soaking the scaffolding	- histological analysis (SEM) - X-rays	Groups with IGF-1 and BMP-2: - A higher density of newly formed bone was observed at the healing site in groups using scaffolds containing growth factors. - greater accumulation of erythrocytes, osteoblasts and mononuclear cells - more intense angiogenesis - normal bone appears faster with more Haversian systems
Sularsih [82]	In vivo, 36 male Cavia cobaya (guinea pigs), aged 3–3.5 months	chitosan–aloe vera scaffold	VEGF	SEM, HE and MICONOS, Antibody monoclonal VEGF	The highest VEGF expression and the largest woven alveolar bone were found in the treatment groups with chitosan–aloe vera scaffold.
Yun [63]	In vitro, Human osteosarcoma MG-63 cells	BMP-2/Hep-Chitosan scaffolds	BMP-2- scaffolds incubated in MES buffer containing heparin and EDC for 24 h at 4 °C, immobilization of BMP-2 for 4 h. Sterilization in 70% ethanol and washing with PBS	SEM, ELISA, ALP, Ca deposition, RT-PCR	The expression levels of osteocalcin and osteopontin were upregulated in the BMP-2 (100 ng)/Hep-chitosan group Cell proliferation higher in BMP-2 (100 ng)/chitosan scaffold
Guzman [78]	In vitro: C2C12 cell line In vivo: 20 New Zealand male rabbits of ca. 3 kg in weight	CHI, CPS-CHI, rhBMP2-CHI, and rhBMP2-CPS-CHI scaffolds	CPS, BMP-2—Urease-assisted preparation of chitosan hydrogels, ISISA process	SEM, Invitrogen, ALP, ELISA, Micro-CT, Histology and histomorphometry	Cells proliferation and osteoinduction higher in scaffolds with both CPS and rhBMP-2.
De la Riva [84]	In vivo New Zealand rabbits, male Femur defects In vitro-release kinetics of GF	Brushite–chitosan scaffold	PDGF—dissolved in liquid phase of brushite, VEGF—encapsulated in alginate microspheres inside chitosan sponges,	Bone regeneration was assessed using in vivo methods: - Histology (Goldner’s Trichrome), - Histomorphometry (ROI), - Mineral apposition rate (MAR) measured by fluorochrome labeling (tetracycline + calcein)	Osteogenesis: PDGF increased new bone formation and osteoblastic activity VEGF enhanced later-stage bone formation PDGF + VEGF combination led to the greatest bone area, larger trabeculae, and more osteoblast layers. Mineralization: Greater mineralized bone and osteoid matrix were observed, especially in the dual-factor group (VP). Cellular activity: Mitotic osteoprogenitor cells and multilayered osteoblasts were observed in the VEGF + PDGF group. Angiogenesis: Not directly measured.
Demirtaş [81]	In vitro Cell culture: MC3T3-E1 mouse pre-osteoblast cell line	Chitosan-based scaffold	- PDGF-BB—onto crosslinked gelatin microparticles - BMP-6—encapsulated in submicron particles of PHBV (poly 3-hydroxybutyric acid-co-3-hydroxyvaleric acid)	RT-PCR for gene expression of osteogenic markers: RunX2, Col1(Collagen type I), OPN (Osteopontin), OCN (Osteocalcin). SEM imaging MTT assay Calcium deposition observed via SEM/EDAX.	Osteogenesis: higher expression of RunX2, Col1, OPN, and OCN, especially with dual factor delivery, Cell proliferation: markedly increased on PDGF-BB-containing scaffolds, Mineralization: calcium deposits detected after 7 days in scaffolds with both growth factors, Cell attachment and morphology: improved spreading and cell–cell interaction observed on dual-loaded scaffolds. Angiogenesis: Not directly measured in this study.
Xie [80]	In vitro and in vivo BMSCs—bone marrow stromal cells Sprague-Dawley rats	OCP-GO/CS scaffolds (octacalcium phosphate mineralized graphene oxide/chitosan scaffolds)	BMP-2-encapsulated BSA (bovine serum albumin) nanoparticles (CBB-NPs)	- ALP activity assay - BMSC proliferation assays - Histological staining (Hematoxylin and Eosin, and Masson’s Trichrome) in a cranial defect model	BMP-2 had a significant effect on promoting osteogenic differentiation, as evidenced by elevated ALP activity. It also enhanced BMSC proliferation and supported new bone formation in vivo. The study did not directly evaluate effects on angiogenesis or calcium deposition separately.
Qiu [79]	In vitro—BMSCs—bone marrow mesenchymal stem cells, rats In vivo—SD rats, calvarial defect model—osteogenic effect	silk fibroin (SF) and chitosan (CS)	BMP-2 was immobilized using mesoporous hydroxyapatite nanoparticles (mHANPs).	- Osteogenic marker gene expression (e.g., Runx2, ALP, Collagen I, Osteocalcin) - Calcium deposition via Alizarin Red S staining - Histological staining (H&E, Masson’s Trichrome) - X-ray imaging	BMP-2 significantly enhanced osteogenic differentiation, matrix mineralization, and cell attachment and proliferation in vitro. In vivo, BMP-2 promoted new bone formation within rat cranial defects. Angiogenesis was not evaluated in this study.

**Table 4:** Quality assessment—JBI checklist for quasi-experimental studies (nonrandomized experimental studies).

Authors	1. Is It Clear in the Study What Is the ‘Cause’ and What Is the ‘Effect’?	2. Were the Participants Included in Any Comparisons Similar?	3. Were the Participants Included in Any Comparisons Receiving Similar Treatment/Care, Other than the Exposure or Intervention of Interest?	4. Was There a Control Group?	5. Were There Multiple Measurements of the Outcome Both Pre and Post the Intervention/Exposure?	6. Was Follow Up Complete and If Not, Were Differences Between Groups in Terms of Their Follow Up Adequately Described and Analyzed?	7. Were the Outcomes of Participants Included in Any Comparisons Measured in the Same Way?	8. Were Outcomes Measured in a Reliable Way?	9. Was Appropriate Statistical Analysis Used?
Wang et al. [54]	yes	yes	yes	yes	yes	yes	yes	yes	yes
Yun et al. [62]	yes	yes	yes	yes	yes	yes	yes	yes	yes
Sanjaya et al. [76]	yes	yes	yes	yes	no	yes	yes	yes	yes
Tigli et al. [85]	yes	yes	yes	yes	yes	yes	yes	yes	yes
Liu et al. [83]	yes	yes	yes	yes	yes	yes	yes	yes	yes
Barakzai et al. [56]	yes	yes	yes	yes	yes	yes	yes	yes	yes
Chen [51]	yes	yes	yes	yes	yes	yes	yes	yes	yes
Soriente [52]	yes	yes	yes	yes	yes	yes	yes	yes	yes
Soran [55]	yes	yes	yes	yes	yes	yes	yes	yes	yes
Nandi [77]	yes	yes	yes	yes	yes	yes	yes	yes	yes
Sularsih [82]	yes	yes	yes	yes	yes	yes	yes	yes	yes
Yun [63]	yes	yes	yes	yes	yes	yes	yes	yes	yes
Guzman [78]	yes	yes	yes	yes	yes	yes	yes	yes	yes
De la Riva [84]	yes	yes	yes	no	yes	yes	yes	yes	yes
Demirtaş [81]	yes	yes	yes	yes	yes	yes	yes	yes	yes
Xie [80]	yes	yes	yes	yes	yes	yes	yes	yes	yes
Qiu [79]	yes	yes	yes	no	yes	yes	yes	yes	yes

## Data Availability

No new data were created or analyzed in this study. Data sharing is not applicable to this article.
